# Soil Microbial Fuel Cell Based Self-Powered Cathodic Biosensor for Sensitive Detection of Heavy Metals

**DOI:** 10.3390/bios13010145

**Published:** 2023-01-15

**Authors:** Shi-Hang Wang, Jian-Wei Wang, Li-Ting Zhao, Syed Zaghum Abbas, Zhugen Yang, Yang-Chun Yong

**Affiliations:** 1 Biofuels Institute, Jiangsu Collaborative Innovation Center of Technology and Material of Water Treatment, School of Environment and Safety Engineering, Zhenjiang 212013, China; 2 School of Water, Environment and Energy, Cranfield University, Milton Keynes MK43 0AL, UK

**Keywords:** soil microbial fuel cells, heavy metals, biosensors, soil pollution

## Abstract

Soil microbial fuel cells (SMFCs) are an innovative device for soil-powered biosensors. However, the traditional SMFC sensors relied on anodic biosensing which might be unstable for long-term and continuous monitoring of toxic pollutants. Here, a carbon-felt-based cathodic SMFC biosensor was developed and applied for soil-powered long-term sensing of heavy metal ions. The SMFC-based biosensor generated output voltage about 400 mV with the external load of 1000 Ω. Upon the injection of metal ions, the voltage of the SMFC was increased sharply and quickly reached a stable output within 2~5 min. The metal ions of Cd^2+^, Zn^2+^, Pb^2+^, or Hg^2+^ ranging from 0.5 to 30 mg/L could be quantified by using this SMFC biosensor. As the anode was immersed in the deep soil, this SMFC-based biosensor was able to monitor efficiently for four months under repeated metal ions detection without significant decrease on the output voltage. This finding demonstrated the clear potential of the cathodic SMFC biosensor, which can be further implemented as a low-cost self-powered biosensor.

## 1. Introduction

Currently, water pollution caused by heavy metals has attracted great attention from all over the world because water is basic source of life. The contamination of water bodies, especially by heavy metals, is a serious threat to human health since the heavy metals cannot be degraded in nature and easily accumulate in the human body [1,2]. Excessive accumulation of heavy metals can lead to the impairment of the human central nervous system, the reduction of energy levels, and the destruction of blood components, lungs, kidneys, liver, and other important body organs [3]. Moreover, water bodies are also vulnerable to heavy metals pollution caused by anthropogenic sources such as industrial waste or natural leakage from the metal minerals [4]. Therefore, it is very important to develop in situ and long-term stable water alarm sensors to monitor the heavy metals in the water body.

Traditional detection methods, such as spectrophotometry and atomic absorption spectrometry (AAS), have high selectivity and sensitivity, but they cannot be used for in situ monitoring because of their high cost and complicated sample pretreatment requirements [5]. Recently, microbial fuel cells (MFCs) have attracted much attention due to their characteristics of energy saving, low cost, easy operation, and sustainability [6]. MFCs use electroactive bacteria (EAB) as biocatalysts to convert chemical energy of organic substrates into electrical energy [7,8,9,10]. By metabolizing organic matter with bacteria, electrons are released (Equation (1)) and transferred to the anode, where the electrons are further passed through the external circuit to the cathode and undergo a reduction reaction with the electron acceptor (e.g., O_2_) (Equation (2)) [11].

At anode: (CH_2_O)_n_ + H_2_O → CO_2_ + H^+^ + e^−^ (1)

At cathode: O_2_ + 4H^+^ + e^−^→ 2H_2_O (2)

More recently, MFCs have been explored for the development of self-powered biosensing systems. However, most of these MFC-based biosensors are bioanodic sensors. The contaminated water in the anodic chamber inhibits metabolic activities of EAB, resulting in decreased electrons generation and lower electrical signal, which is then used as the indicator for water toxicity detection [12]. However, one major disadvantage of such biosensors is that they have difficulty with long-term and repeated monitoring, because toxic pollutants can inhibit the activity of EAB, and the high concentration of pollutants even causes irreversible damage, which renders the biosensor unable to operate stably for a long time and limits the practical application of bioanodic sensors [13]. Thus, for long-term and stable monitoring of MFC-based biosensors, it is unrealistic to use anodes as sensing elements.

EAB are commonly found in aquatic environments such as swamps, lakes, and marine sediments [14,15,16,17]. SMFCs can consume abundant organic matter in soil to generate electrical signals by using EAB long-term [18]. The SMFC consists of an anode buried in the soil and a cathode adjusted in the overlying water [19]. Since most of the heavy metal solutions are acidic, the water containing heavy metal ions reduces the pH value near the cathode, increasing the oxidation–reduction potential (ORP) of the overlying water and acting as an electron acceptor, promoting the cathodic reaction rate; ultimately, the SMFC-based biosensor generates response signals [20,21]. At the same time, the toxic effect of heavy metals on EAB is limited because soil absorbs heavy metal ions and reduces their toxicity. The SMFC-based biosensor can run stably for a long time [22]. However, the cathodic SFMC biosensors reported usually use platinum as the cathode, which is high in cost and limits its practical application. It is still unclear whether low-cost carbon electrodes can be used for cathodic SMFC-based sensors.

In this study, a carbon felt cathode SMFC-based biosensor was developed, which not only generated power from soil but also repeatedly monitored the heavy metal ions of Cd^2+^, Zn^2+^, Pb^2+^, and Hg^2+^ in the water for 4 months. During 4 months of repeated heavy metals detection, the SMFC showed relatively stable voltage output, while also exhibiting reliable analytical performance towards different samples. Further, the underlying mechanism for the cathodic response to the heavy metals was also explored. The development of a carbon felt cathodic SMFC-based biosensor in this study might promote the practical application of low-cost self-powered SMFC-based biosensors.

## 2. Materials and Methods

### 2.1. Sampling of Soil

The sediment soil was taken from the sediments of the lake in the campus of Jiangsu University, China (N 32°11′35″, E 119°35′37″), at a depth of 30–40 cm. After sampling, large particles in the soil were removed with a 2 mm mesh and settled naturally for two days.

### 2.2. SMFC Sensor Setup and Operation

**Figure 1:**
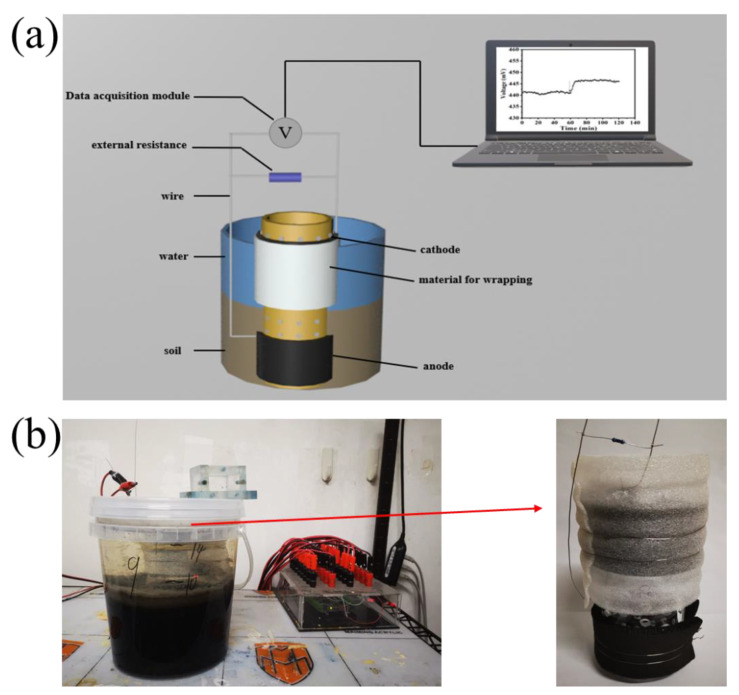
The schematic diagram (**a**) and prototype (**b**) of the SMFC-based sensor. The red arrow in (**b**) indicates the anode and cathode assembly of the SMFC.

Soil was placed in a Ø 15 cm × 17 cm (diameter × height) PE bucket with a soil layer height of 7–9 cm, then the lake water was added into a PE bucket where the water level was 5–7 cm above the soil surface. Each biosensor consists of an electrode material, an electrode wrapping material, and a porous polymethyl methacrylate (PMMA) tube. Carbon felt (TW-YB, carbon energy, Taiwan) was selected as the electrode material for anode and cathode, the size of each was 15 cm × 4 cm (length × height), and the thickness of carbon felt was 0.5 cm. The porous polymethyl methacrylate (PMMA) tube was Ø 6 cm × 18 cm in size, the tube wall was uniform with holes (Ø 0.5 cm), and the electrode wrapping material was pearl cotton. The anode and cathode were connected to a 1000 Ω external resistor with titanium wires. The anode was buried in the flooded soil to collect the electrons produced by natural EAB, while the cathode was immersed in the covering water using dissolved oxygen as the electron acceptor. A data collector (MPS-010602, QIChuang Mofei Electronic Technology, Beijing, China) was used to record the output voltage across the 1000 Ω external resistor every minute (Figure 1).

### 2.3. Heavy Metal Ions Detection

After 30 days’ domestication, four SMFCs with stable voltage output were used as the biosensors for heavy metal ions detection. During the four-month operation, heavy metal solutions of CdCl_2_, ZnCl_2_, PbCl_2_, or HgCl_2_ were added to SMFC-based biosensors. To avoid cross-talk between different heavy metals, one SMFC-based biosensor was only used to detect the selected ions.

When the output voltage was stable, the data collector was used to record the voltage data for one hour. Then, the final concentrations of 0.5, 1, 5, 10, 20, 30, 40, and 50 mg/L of Cd^2+^ were added to the surface water and then the voltage data were recorded for an hour. The detection method of Zn^2+^, Pb^2+^, or Hg^2+^ ions was the same as that for Cd^2+^.

The voltage signals difference before and after the heavy metal addition was used as the heavy metal response signal. The average value of the voltage until 20 min, 10 min, and 0 min before the injection of heavy metal solution was denoted as the baseline voltage. The average value of the voltage until 10 min, 20 min, and 30 min after the injection of heavy metal solution is denoted as the peak voltage. The voltage increment (ΔV) as the response of the SMFC-based biosensor was calculated as the following:

ΔV = V_p_ – V_b_ (3)

Here, V_p_ is the peak voltage after the addition of heavy metal solution and V_b_ is the baseline voltage before the addition of heavy metal solution.

The detection of specific ion was operated repeatedly in one SMFC (without medium or electrode amendment or replacement) for the whole biosensing process from day 30 to day 100, while different ions used different SMFCs.

### 2.4. Electrochemical and Physical Analysis

Electrochemical impedance spectroscopy (EIS) was performed using an electrochemical workstation (CHI660E, Chenhua, Shanghai, China). A three-electrode system was constructed to detect the charge transfer resistance of the cathode. The saturated calomel electrode (CHI150, Chenhua, Shanghai, China) was used as the reference electrode. The EIS was operated in the frequency range of 10 mHz~100 kHz with an amplitude of 10 mV. The Nyquist diagram was fitted to the equivalent circuit using ZView software. After the heavy metals were detected by the SMFC-based biosensor, four groups of SMFC cathode carbon felt (0.25 cm^2^) were cut and their elemental and chemical states on the cathode surface were characterized by an X-ray photoelectron spectrometer (PHI-1600, PerkinElmer, Waltham, MA, USA). The concentrations of ICP in water samples were also quantified with ICP-MS (ICPE-9000, Shimadzu, Japan) using the method described elsewhere [23,24].

### 2.5. High-throughput 16S rRNA Gene Sequencing

PCR amplification, PCR product purification and quantification, and sequencing were conducted with the Illumina MiSeq platform (Shanghai Sangon Biotech Co., Ltd., Shanghai, China). Amplicon libraries were constructed using bacterial universal primers 341 F (5′-CCTACGGGNGGCWGCAG-3′) and 805 R (5′-GACTACHVGGGTATCTAATCC-3′). Data analysis was performed as described elsewhere [25,26].

## 3. Results and Discussion

### 3.1. Bioelectricity-Producing Performance of the SMFC

**Figure 2:**
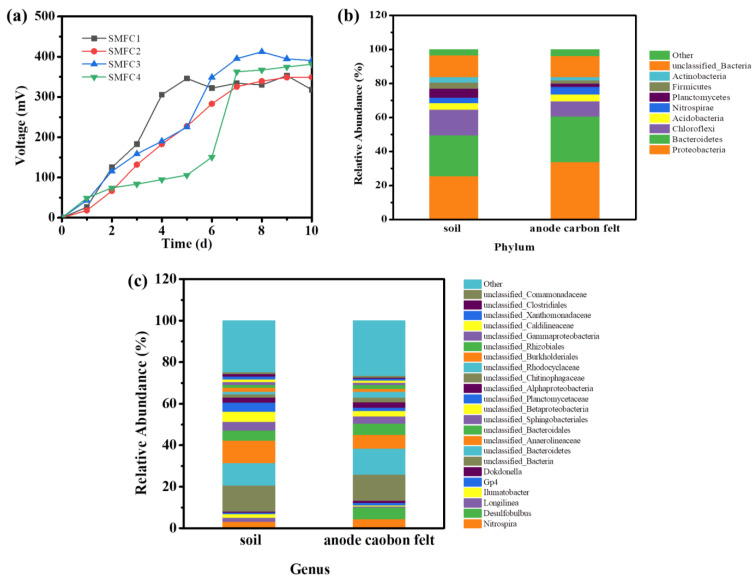
The voltage output of the four SMFCs (**a**) and the microbial community analysis of the anodic biofilm of the SMFC (**b**,**c**).

The SMFCs equipped with the carbon felt as the anode and cathode were constructed by using the lake sediment as the inoculum. Upon the incubation of the SMFC under 30 °C, the voltage output was gradually increased and reached the steady voltage of about ~330–400 mV over 1000 Ω resistor (Figure 2a). The open circuit voltage (OCV) for SMFC1–SMFC3 was over 750 mV (Figure S1). Next, the power output of the SMFCs was evaluated by measuring the polarization curves. The highest power of 54 μW among these four SMFCs was obtained (Figure S1). These results indicated that the SMFCs were successfully constructed.

Then, the MiSeq sequencing of the 16S rRNA gene was used to analyze the diversity of the microbial community of the anode biofilm (Figure 2b). It was found that, after one month of SMFC operation, the portion of the Proteobacteria in the anode biofilm was increased obviously compared with the original soil. It was reported that a lot of soilborne EAB belong to Proteobacteria, such as *Pseudomonas aeruginosa* and *Geobacter* Spp [27]. As shown in Figure 2b, Proteobacteria accounted for 33.87% of the total number of microorganisms on the anodic carbon felt, which was 1.33 times that in the original soil (25.54%). Bacteroidetes, another main phylum in the SMFC anode, was also increased compared to that of the original soil [28]. Further, according to the analysis at the genus level, it was found that *Desulfobulbus* was dramatically enriched in the anodic biofilm (Figure 2c). As reported previously, the electrogenic bacteria species belongs to *Desulfucapsa*, *Desulfobulbus,* or *Desulfuromonas acetoxidans,* which are usually enriched in the biofilm of different SMFCs [18,29]. All these results indicated that the electrogenic bacteria were enriched in the anodic biofilm and further confirmed that the SMFCs were successfully constructed.

### 3.2. Development of Carbon Felt Cathodic Sensing System with SMFC

**Figure 3:**
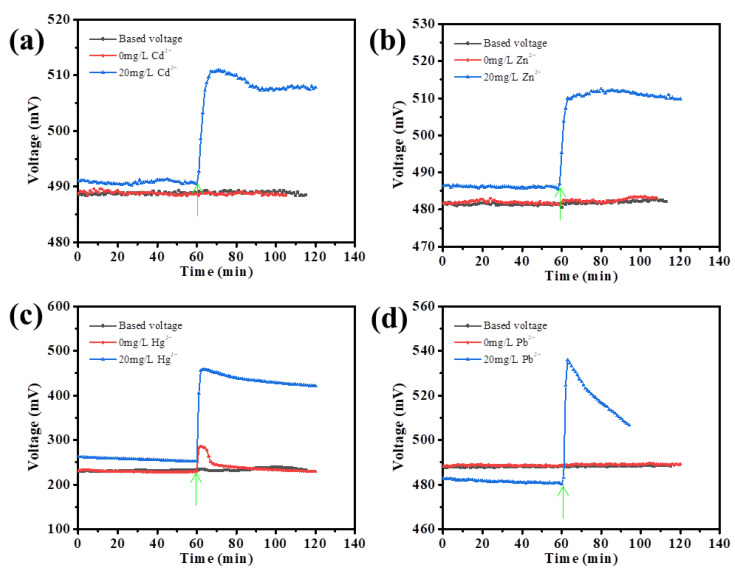
The response of the SMFC voltage output to the addition of Cd^2+^ (**a**), Zn^2+^ (**b**), Hg^2+^ (**c**), or Pb^2+^ (**d**). The green arrow indicates the injection of the heavy metal ions.

To develop the cathodic sensing system with the SMFC, the response of the voltage output to the addition of different heavy metal ions was tested. It could be observed that after 30 days operation, these four SMFCs reached steady voltage output (Figure 3). It was speculated that they were ready for sensing system construction. As shown in Figure 3a, upon the addition of 20 mg/L Cd^2+^, the voltage output suddenly increased and reached the highest output in about 7 min, while the voltage increased from ~490 mV to ~510 mV. The net voltage increment was about 20 mV. More impressively, the voltage output further reached the high and stable value of about 507 mV. The results substantiated that this cathodic sensing mode did not significantly influence the electricity-generation capacity of the anode. For the Zn^2+^ addition, the output voltage was increased from 486 mV to 511 mV in ~6 min, which was reached the stable value of ~511 mV (Figure 3b). The net voltage output increment was ~25 mV, even higher than that induced by the Cd^2+^ addition. Next, the effect of more toxic heavy metal of Hg^2+^ or Pb^2+^ on the voltage output of the SMFCs was evaluated (Figure 3c,d). As shown in Figure 3c, upon the addition of 20 mg/L Hg^2+^, the voltage output was quickly increased from ~252 mV to ~458 mV in about 2 min. The net voltage increment was about 206 mV, which was much higher than that from the Cd^2+^ or Zn^2+^. It also reached a relatively stable output of about 425 mV. For Pb^2+^ detection, as it easily participated under alkaline condition, the sample solution was adjusted to a low pH of about 4. After addition of the Pb^2+^ solution, the voltage of the SMFC also quickly increased (Figure 3d). Upon the addition of 20 mg/L Pb^2+^, the voltage output was quickly increased from ~480 mV to ~536 mV in about 3 min. The net voltage increment was about 56 mV. However, the voltage quickly decreased, as described by other groups [13,27]. All the results indicated that the addition of different heavy metals in the cathodic part did not significantly impair the bioelectricity generation capacity, while the voltage output quickly increased in response to the heavy metal addition. These results suggest that the SMFC might be adopted as the self-powered sensing system for the detection of heavy metals.

### 3.3. Analytical Performance of the SMFC Self-Powered Biosensing System

Next, the analytical performance of the SMFC-based self-powered biosensing system was determined. Firstly, the SMFC responses to different concentrations of the heavy metal were recorded. It was found that the SMFC showed a significant response to Cd^2+^ from the concentration of 1 mg/L (Figure S2). The highest voltage increment of 54.9 ± 3.3 mV was recorded with the addition of 40 mg/L of Cd^2+^, while the voltage increment became lower after the addition of Cd^2+^ over 50 mg/L. For Zn^2+^, the response became obvious from the concentration of 1 mg/L, while the highest voltage increment of 34.9 ± 2.3 mV occurred at the concentration of 30 mg/L (Figure S3). For Hg^2+^, it was observed that obvious response occurred from 0.1 mg/L to 30 mg/L, while the highest voltage increment at 30 mg/L was 211.1 ± 0.5 mV (Figure S4). For Pb^2+^, the significant response was observed from the concentration of 0.5 mg/L (Figure S5), while the highest voltage increment of 54.6 ± 2.1 mV occurred at the concentration of 30 mg/L. These results substantiated that the output of the SMFC-based sensing system showed dose-dependent responses to different heavy metals, which implies that quantification of these heavy metals might be possible with this system.

**Figure 4:**
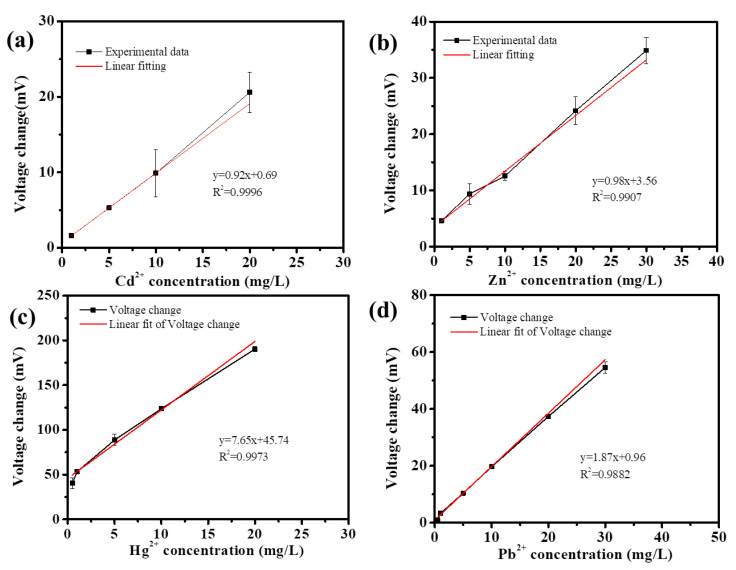
The calibration curves for Cd^2+^ (**a**), Zn^2+^ (**b**), Hg^2+^ (**c**), or Pb^2+^ (**d**) as detected by the developed SMFC-based sensing system.

Thus, the calibration curves for different heavy metal ions were determined. As shown in Figure 4, a linear relationship between the voltage increment and the concentration of Cd^2+^, Zn^2+^, Hg^2+^, or Pb^2+^ was obtained. It was found that the linear correlation coefficients were 0.999, 0.991, 0.997, and 0.988 for Cd^2+^, Zn^2+^, Hg^2+^, or Pb^2+^, respectively. The linear ranges were 1–20 mg/L, 1–30 mg/L, 0.5–30 mg/L, and 0.5–20 mg/L, respectively (Figure 4). The representative SMFC-based sensing systems for detection of heavy metals reported in the recent years are summarized in Table 1. Compared with other cathodic SMFC-based sensing systems, the sensing system developed here showed the advantages of low-cost cathode material or wider linear detection range. Moreover, although repeated detection of heavy metal ions was applied (from 30 to 100 days) during the operation, these four SMFCs still maintained relatively stable electricity generation (Figure S6).

Then, the quantification of Pb^2+^ in water samples by this self-powered SMFC-based sensing system was demonstrated. Five different samples with different concentrations of Pb^2+^ were quantified by the sensing system and compared with the results obtained with the ICP. It was clear that the coefficients of variation between these two methods were lower than 9% (Table 2), implying that it was reliable to use this developed sensing system for heavy metal quantification. However, as the real-life environment and conditions were more complicated, more sophisticated design in consideration of the real samples and in-field test would be desirable before its practical implementation.

### 3.4. Underlying Mechanism for the Cathodic Sensing of SMFC

**Figure 5:**
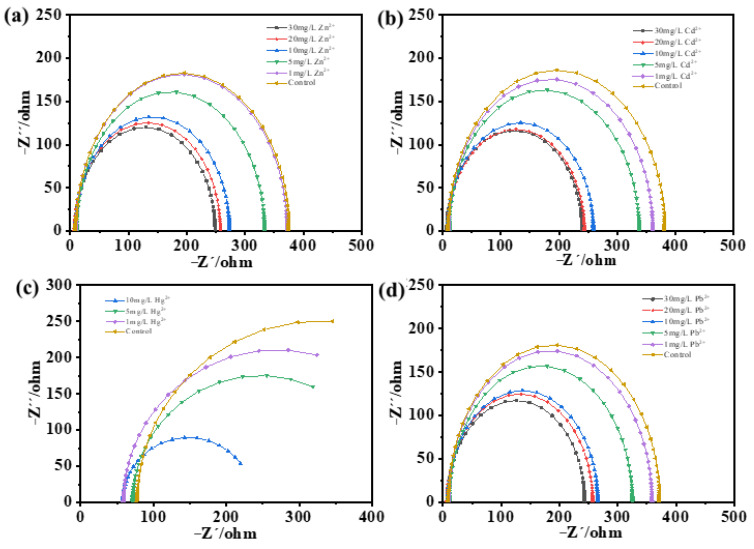
The EIS spectrum of the SMFCs after the detection of Cd^2+^ (**a**), Zn^2+^ (**b**), Hg^2+^ (**c**), or Pb^2+^ (**d**).

Furthermore, the underlying mechanism for the cathodic sensing was discussed. It was speculated that the voltage increments upon the addition of heavy metal ions might be due to the cathodic reducing of the metal ions to zero-valent metals. The EIS was measured one hour after the addition of Cd^2+^, Zn^2+^, Pb^2+^, or Hg^2+^, respectively. The Nyquist plot of the cathode is shown in Figure 5. Obviously, with the increase of the concentration of Cd^2+^, Zn^2+^, Pb^2+^, or Hg^2+^, the diameter of the semicircle was decreased. The diameter of the semicircle indicated the charge transfer resistance (R_ct_), which was an indicator of the electrochemical reaction rate of the electrode surface [33]. The decrease of R_ct_ indicated that the addition of heavy metal ions promoted the cathodic reaction rate.

**Figure 6:**
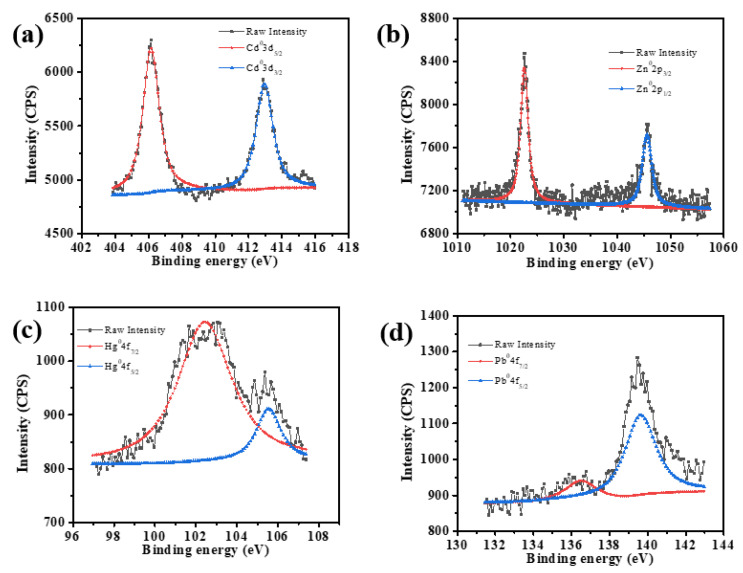
The XPS spectrum of the SMFCs cathode after the detection of Cd^2+^ (**a**), Zn^2+^ (**b**), Hg^2+^ (**c**), or Pb^2+^ (**d**).

XPS was further applied to analyze the chemical state of the heavy metals on the cathode surface after biosensing (Figure 6). The results showed that a Cd^0^3d_5/2_ peak appeared at the binding energy of 405 eV [34], a Zn^0^2p_3/2_ peak appeared at the binding energy of 1022.5 eV [35], a Pb^0^4f_7/2_ peak appeared at the binding energy of 136.6 eV [36], and a Hg^0^4f7/2 peak appeared at the binding energy of 102.3 eV. The results indicated that Cd^2+^, Zn^2+^, Pb^2+^, and Hg^2+^ were reduced as electron acceptors and deposited on the cathode surface in the form of Cd(0), Zn(0), Pb(0), and Hg(0). Therefore, it can be concluded that the addition of Cd^2+^, Zn^2+^, Pb^2+^, or Hg^2+^ provided electron acceptors, promoted the cathodic reaction, and resulted in the voltage increment, which would be the underlying mechanism for the SMFC-based cathodic sensing heavy metal ions.

## 4. Conclusions

In this study, a carbon felt cathode SMFC-based sensing system for the detection of four typical heavy metal ions was developed. This SMFC-based sensing system was continuously operated efficiently for 4 months. This self-powered SMFC-based sensing system can use heavy metal ions such as Cd^2+^, Zn^2+^, Pb^2+^, or Hg^2+^ as the electron acceptor and shows dose-dependent response with a relatively wide concentration range. This work demonstrates that low-cost carbon felt could be used as the cathode for the development of SMFC-based cathodic sensing systems, which would be promising for the development of in situ and long-term monitoring devices for the remote areas that are short of electric power.

## Figures and Tables

**Table 1:** Comparison of the analytical performance of representative MFC-based sensing systems reported recently.

Configuration	Sensing Element	Electrode Material	Heavy Metals	Electric Change	MFC Voltage	Linear Range for Detection	Sensitivity (Estimated) (mV/mg)	References
Dual-chamber MFC	Bioanode	Anode, carbon felt; cathode, CuO/ZnO	Cu^2+^, Cd^2+^	Decrease	200 mV	Cd^2+^ 0.1–4 mg/L Cu^2+^ 10–80 mg/L	-	[30]
Dual-chamber MFC	Bioanode	Anode, carbon felt; cathode, carbon felt	Zn^2+^	Increase	250 mV	Zn^2+^ 20–100 μM	~0.5	[31]
Single-chamber MFC	Bioanode	Anode, graphite; cathode, graphite	Cu^2+^,Cr^6+^, Zn^2+^, Ni^2+^	Decrease	200 mV	Cu^2+^ 5–20 mg/L Cr^6+^ 5–20 mg/L Zn^2+^ 5–20 mg/L Ni^2+^ 5–20 mg/L	Cu^2+^ ~4.5 Cr^6+^ ~6.5 Zn^2+^ ~5.0 Ni^2+^ ~4.0	[32]
Single-chamber MFC	Cathode	Anode, stainless steel; cathode, platinum	Cu^2+^	Increase	~170 mV	Cu^2+^ 12.5–400 mg/L	~0.1–0.3	[22]
Single-chamber MFC	Cathode	Anode, stainless steel; cathode, platinum	Cu^2+^	Increase	~200 mV	Cu^2+^ 0.3–2.5 mg/L	~0.1	[13]
Single-chamber MFC	Cathode	Anode, carbon felt; cathode, carbon felt	Cd^2+^, Zn^2^, Pb^2+^, Hg^2+^	Increase	~42,700 mV	Cd^2+^ 1–30 mg/L Zn^2+^ 1–30 mg/L Pb^2+^ 0.5–30 mg/L Hg^2+^ 0.5–20 mg/L	Cd^2+^ ~0.92 Zn^2+^ ~0.98 Pb^2+^ 1.87 Hg^2+^ 7.65	This study

**Table 2:** Quantification of Pb^2+^ in different lake water samples with SMFC sensing system and ICP.

Sample	Detected by SMFC (mg/L)	Detected by ICP (mg/L)	Coefficient of Variation (%)
Sample 1	3.58	3.64	−1.68
Sample 2	5.28	5.26	0.38
Sample 3	8.11	8.49	−4.75
Sample 4	12.81	13.50	−5.39
Sample 5	16.70	18.20	−8.98

## Data Availability

Data are present within the article.

## Supplementary Materials

The following supporting information can be downloaded at: https://www.mdpi.com/article/10.3390/bios13010145/s1, Figure S1: The power output (a) and polarization curves (b) of these four SMFCs; Figure S2: The response of SMFC based sensing system to the addition of Cd^2+^ at different concentrations; Figure S3: The response of SMFC based sensing system to the addition of Zn^2+^ at different concentrations; Figure S4: The response of SMFC based sensing system to the addition of Hg^2+^ at different concentrations; Figure S5: The response of SMFC based sensing system to the addition of Pb^2+^ at different concentrations; Figure S6: The long-term voltage output of these four SMFCs.

## References

[1] Gumpu M.B., Sethuraman S., Krishnan U.M., Rayappan J.B.B. (2015). A review on detection of heavy metal ions in water—An electrochemical approach. Sens. Actuators B Chem..

[2] Yang Y., Fang Z., Yu Y.-Y., Wang Y.-Z., Naraginti S., Yong Y.-C. (2019). A mediator-free whole-cell electrochemical biosensing system for sensitive assessment of heavy metal toxicity in water. Water Sci. Technol..

[3] Briffa J., Sinagra E., Blundell R. (2020). Heavy metal pollution in the environment and their toxicological effects on humans. Heliyon.

[4] Yu Z., Liu E., Lin Q., Zhang E., Yang F., Wei C., Shen J. (2020). Comprehensive assessment of heavy metal pollution and ecological risk in lake sediment by combining total concentration and chemical partitioning. Environ. Pollut..

[5] Liu S., Wang C., Yang J., Zhao Q. (2014). Assessing the heavy metal contamination of soils in the water-level fluctuation zone upstream and downstream of the Manwan Dam, Lancang River. J. Soils Sediments.

[6] Abourached C., Catal T., Liu H. (2014). Efficacy of single-chamber microbial fuel cells for removal of cadmium and zinc with simultaneous electricity production. Water Res..

[7] Aslan S., Conghaile P., Leech D., Gorton L., Timur S., Anik U. (2017). Development of an Osmium Redox Polymer Mediated Bioanode and Examination of its Performance in Gluconobacter oxydans Based Microbial Fuel Cell. Electroanalysis.

[8] Aslan S., Conghaile P.O., Leech D., Gorton L., Timur S., Anik U. (2017). Development of a Bioanode for Microbial Fuel Cells Based on the Combination of a MWCNT-Au-Pt Hybrid Nanomaterial, an Osmium Redox Polymer and Gluconobacter oxydans DSM 2343 Cells. Chemistryselect.

[9] Moradian J.M., Fang Z., Yong Y.-C. (2021). Recent advances on biomass-fueled microbial fuel cell. Bioresour. Bioprocess..

[10] Yu Y.-Y., Wang Y.-Z., Fang Z., Shi Y.-T., Cheng Q.-W., Chen Y.-X., Shi W., Yong Y.-C. (2020). Single cell electron collectors for highly efficient wiring-up electronic abiotic/biotic interfaces. Nat. Commun..

[11] Zabihallahpoor A., Rahimnejad M., Talebnia F. (2015). Sediment microbial fuel cells as a new source of renewable and sustainable energy: Present status and future prospects. RSC Adv..

[12] Kim M., Sik Hyun M., Gadd G.M., Joo Kim H. (2007). A novel biomonitoring system using microbial fuel cells. J. Environ. Monit..

[13] Wu S., Deng H., Han C., Liu L., Zhong W. (2018). A Novel Sediment Microbial Fuel Cell Based Sensor for On-Line and in situ Monitoring Copper Shock in Water. Electroanalysis.

[14] Deng H., Chen Z., Zhao F. (2011). Energy from Plants and Microorganisms: Progress in Plant-Microbial Fuel Cells. Chemsuschem.

[15] Deng H., Wu Y.-C., Zhang F., Huang Z.-C., Chen Z., Xu H.-J., Zhao F. (2014). Factors Affecting the Performance of Single-Chamber Soil Microbial Fuel Cells for Power Generation. Pedosphere.

[16] Xu F., Mou Z., Geng J., Zhang X., Li C.-Z. (2016). Azo dye decolorization by a halotolerant exoelectrogenic decolorizer isolated from marine sediment. Chemosphere.

[17] Yang Z.-C., Cheng Y.-Y., Zhang F., Li B.-B., Mu Y., Li W.-W., Yu H.-Q. (2016). Rapid Detection and Enumeration of Exoelectrogenic Bacteria in Lake Sediments and a Wastewater Treatment Plant Using a Coupled WO_3_ Nanoclusters and Most Probable Number Method. Environ. Sci. Technol. Lett..

[18] Abbas S.Z., Rafatullah M. (2021). Recent advances in soil microbial fuel cells for soil contaminants remediation. Chemosphere.

[19] Gustave W., Yuan Z.-F., Sekar R., Ren Y.-X., Liu J.-Y., Zhang J., Chen Z. (2019). Soil organic matter amount determines the behavior of iron and arsenic in paddy soil with microbial fuel cells. Chemosphere.

[20] Meunier N., Laroulandie J., Blais J., Tyagi R. (2003). Cocoa shells for heavy metal removal from acidic solutions. Bioresour. Technol..

[21] Wang Z., Deng H., Chen L., Xiao Y., Zhao F. (2013). In situ measurements of dissolved oxygen, pH and redox potential of biocathode microenvironments using microelectrodes. Bioresour. Technol..

[22] Liu L., Lu Y., Zhong W., Meng L., Deng H. (2020). On-line monitoring of repeated copper pollutions using sediment microbial fuel cell based sensors in the field environment. Sci. Total. Environ..

[23] Wen L., Dong J.B., Yang H.S., Zhao J.Y., Hu Z.K., Han H.Y., Hou C.J., Luo X.G., Huo D.Q. (2022). A novel electrochemical sensor for simultaneous detection of Cd2+ and Pb2+ by MXene aerogel-CuO/carbon cloth flexible electrode based on oxygen vacancy and bismuth film. Sci. Total Environ..

[24] Zhao L., Zhong S., Fang K., Qian Z., Chen J. (2012). Determination of cadmium(II), cobalt(II), nickel(II), lead(II), zinc(II), and copper(II) in water samples using dual-cloud point extraction and inductively coupled plasma emission spectrometry. J. Hazard. Mater..

[25] Cui M.-H., Cui D., Liang B., Sangeetha T., Wang A.-J., Cheng H.-Y. (2016). Decolorization enhancement by optimizing azo dye loading rate in an anaerobic reactor. RSC Adv..

[26] Yang C., Liu W., He Z., Thangavel S., Wang L., Zhou A., Wang A. (2015). Freezing/thawing pretreatment coupled with biological process of thermophilic Geobacillus sp. G1: Acceleration on waste activated sludge hydrolysis and acidification. Bioresour. Technol..

[27] Zhao S., Liu P., Niu Y., Chen Z., Khan A., Zhang P., Li X. (2018). A Novel Early Warning System Based on a Sediment Microbial Fuel Cell for In Situ and Real Time Hexavalent Chromium Detection in Industrial Wastewater. Sensors.

[28] Clauwaert P., Aelterman P., Pham T.H., De Schamphelaire L., Carballa M., Rabaey K., Verstraete W. (2008). Minimizing losses in bio-electrochemical systems: The road to applications. Appl. Microbiol. Biotechnol..

[29] Jiang Y.-B., Zhong W.-H., Han C., Deng H. (2016). Characterization of Electricity Generated by Soil in Microbial Fuel Cells and the Isolation of Soil Source Exoelectrogenic Bacteria. Front. Microbiol..

[30] Lu Y., Hu X., Tang L., Peng B., Tang J., Zeng T., Zhang X., Liu Q. (2022). Effect of CuO/ZnO/FTO electrode properties on the performance of a photo-microbial fuel cell sensor for the detection of heavy metals. Chemosphere.

[31] Khan A., Salama E.-S., Chen Z., Ni H., Zhao S., Zhou T., Pei Y., Sani R.K., Ling Z., Liu P. (2019). A novel biosensor for zinc detection based on microbial fuel cell system. Biosens. Bioelectron..

[32] Naik S., Jujjavarapu S.E. (2021). Self-powered and reusable microbial fuel cell biosensor for toxicity detection in heavy metal polluted water. J. Environ. Chem. Eng..

[33] Zhou X., Qu Y., Kim B.H., Choo P.Y., Liu J., Du Y., He W., Chang I.S., Ren N., Feng Y. (2014). Effects of azide on electron transport of exoelectrogens in air-cathode microbial fuel cells. Bioresour. Technol..

[34] Zhang Z., Wang B., Zhou P., Kang R., Zhang B., Guo D. (2016). A novel approach of chemical mechanical polishing for cadmium zinc telluride wafers. Sci. Rep..

[35] Dilasari B., Jung Y., Kwon K. (2017). Effect of water on the stability of zinc in 1-butyl-1-methylpyrrolidinium bis(trifluoromethylsulfonyl)imide ionic liquid. J. Ind. Eng. Chem..

[36] McGettrick J.D., Hooper K., Pockett A., Baker J., Troughton J., Carnie M., Watson T. (2019). Sources of Pb(0) artefacts during XPS analysis of lead halide perovskites. Mater. Lett..

