# Supplementary material for: Soil Microbial Fuel Cell Based Self-Powered Cathodic Biosensor for Sensitive Detection of Heavy Metals
Source: Biosensors (Basel). 2023 Jan 15;13(1):145. doi: 10.3390/bios13010145 (PMC9855947; doi:10.3390/bios13010145)
Supplement: Supplementary file 1 [file biosensors-13-00145-s001.zip › biosensors-2111514-supplementary.pdf]

Supporting Information

Soil microbial fuel cell based self-powered cathodic biosensor for sensitive detection of heavy metals

Shi-Hang Wang [1], Jian-Wei Wang [1], Li-Ting Zhao [1], Syed Zaghum Abbas [1], Zhugen Yang [2] and Yang-Chun Yong [1,*]

[1]Biofuels Institute, Jiangsu Collaborative Innovation Center of Technology and Material of Water Treatment, School of Emergency Management & School of Environment and Safety Engineering, Zhenjiang 212013, Jiangsu Province, China.
[2]School of Water, Environment and Energy, Cranfield University, Milton Keynes MK43 0AL, United Kingdom
*   Correspondence: ycyong@ujs.edu.cn

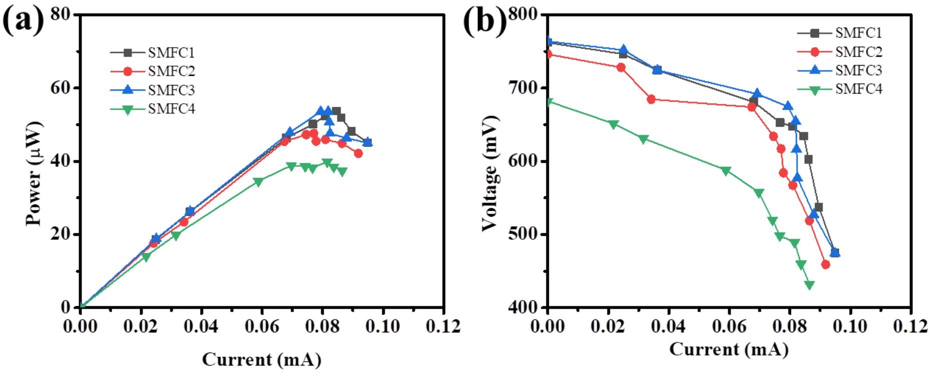

Figure S1. The power output (a) and polarization curves (b) of these four SMFCs.

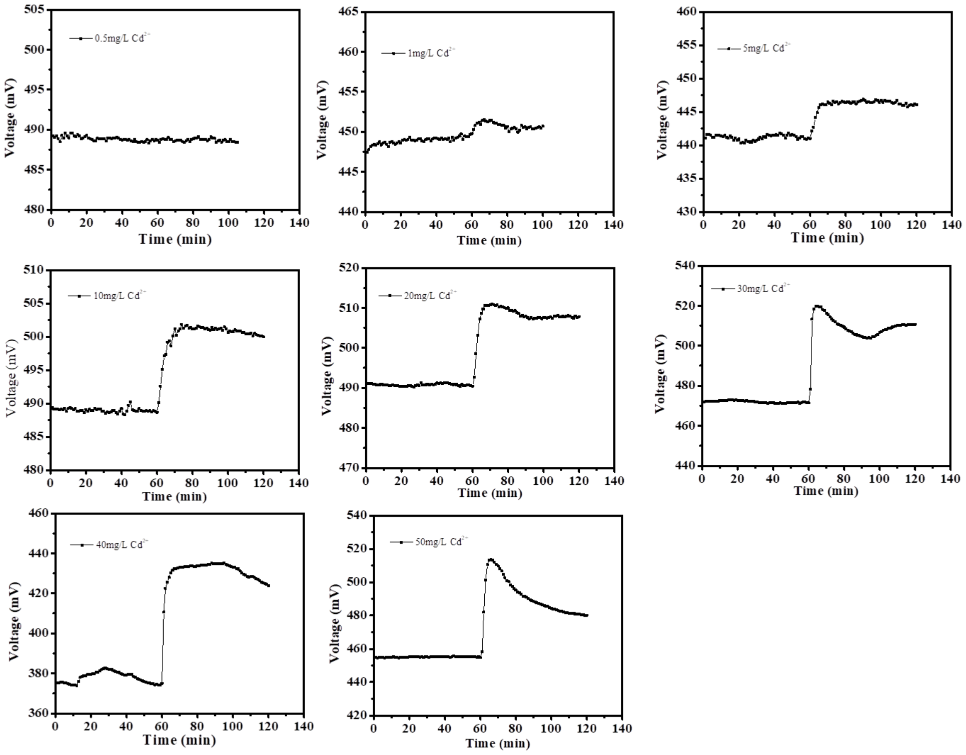

Figure S2. The response of SMFC based sensing system to the addition of Cd^{2+} at different concentrations.

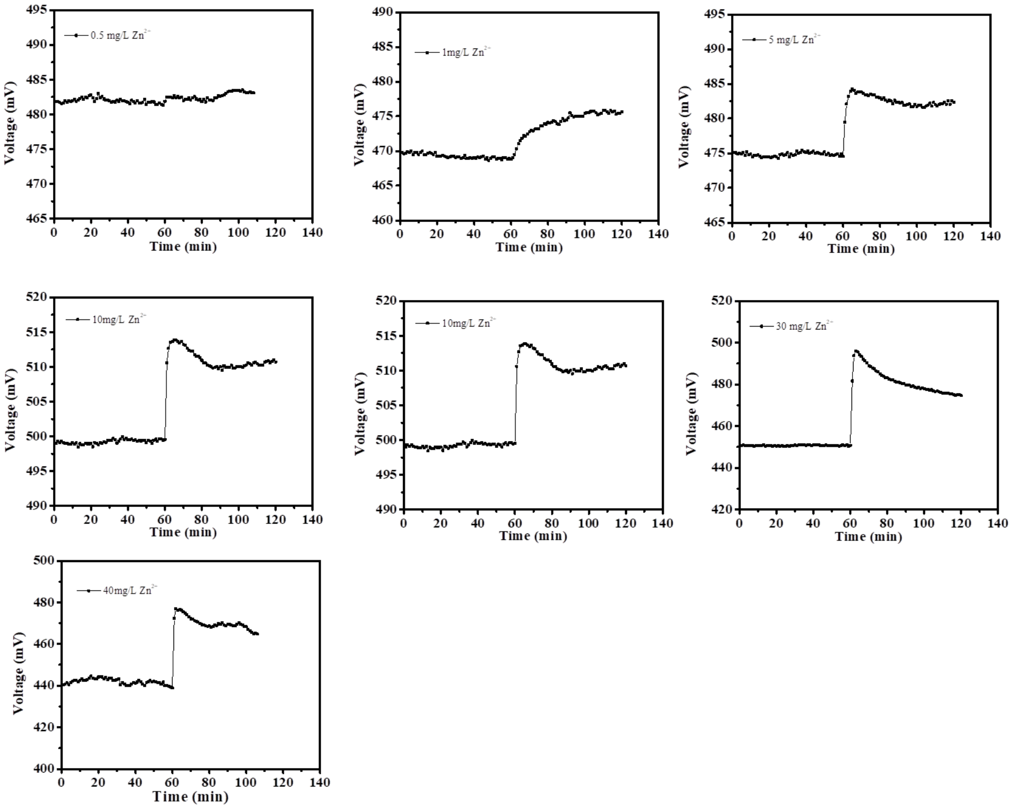

Figure S3. The response of SMFC based sensing system to the addition of Zn^{2+} at different concentrations.

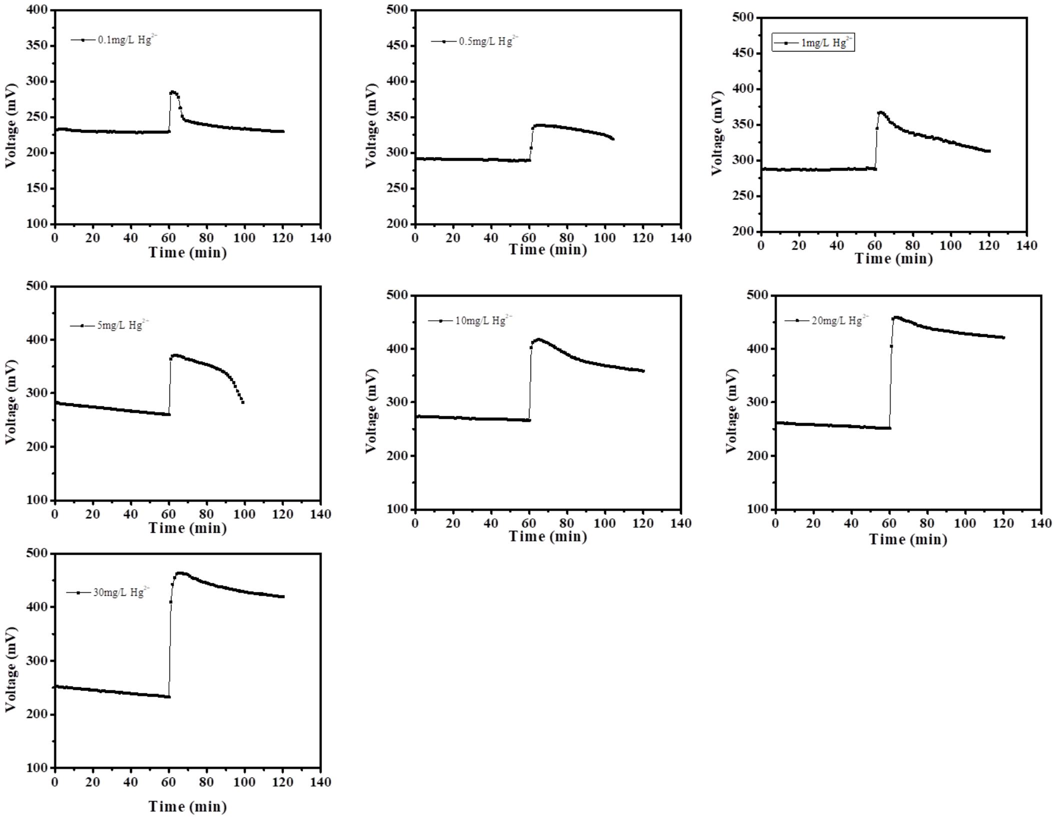

Figure S4. The response of SMFC based sensing system to the addition of Hg^{2+} at different concentrations.

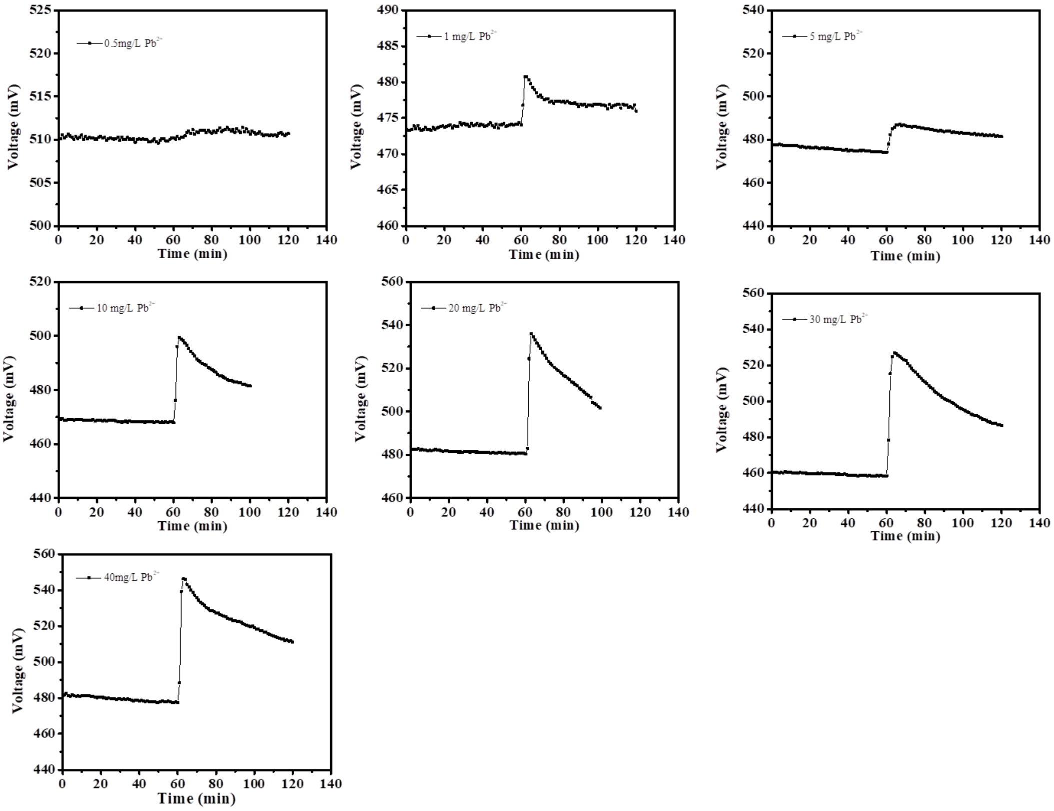

Figure S5. The response of SMFC based sensing system to the addition of Pb^{2+} at different concentrations.

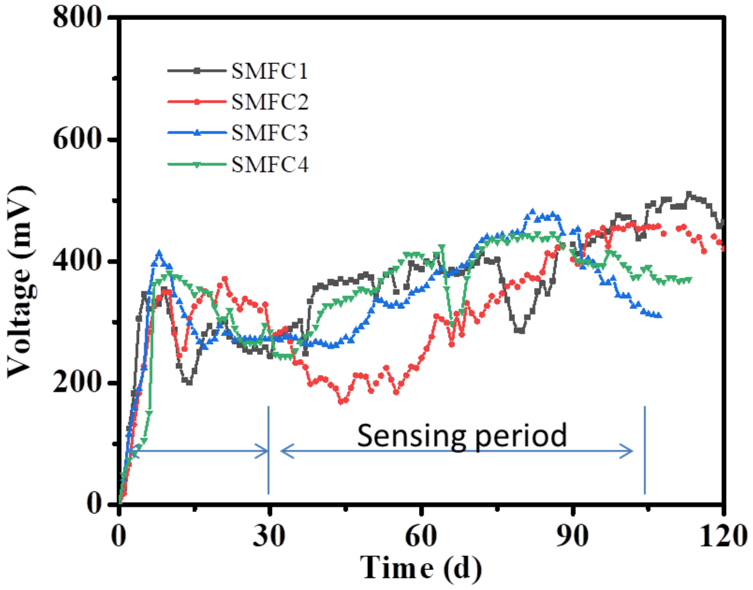

Figure S6. The long-term voltage output of these four SMFCs.
